# Tracheomediastinal Fistula Formation After Endobronchial Ultrasound Transbronchial Needle Aspiration While on Bevacizumab Treatment

**DOI:** 10.7759/cureus.14189

**Published:** 2021-03-30

**Authors:** Alejandra Castro-Varela, Sofia Molina, Horiana B Grosu

**Affiliations:** 1 Department of Pulmonary Medicine, The University of Texas MD Anderson Cancer Center, Houston, USA; 2 School of Medicine and Health Sciences, Tecnológico de Monterrey, Monterrey, MEX

**Keywords:** bevacizumab, tracheoesophageal fistula, respiratory tract fistula, ebus, non-small-cell lung cancer

## Abstract

A 63-year-old male with non-small-cell lung cancer (NSCLC) developed a tracheomediastinal fistula after endobronchial ultrasound transbronchial needle aspiration while on treatment with bevacizumab. This vascular endothelial growth factor-specific angiogenesis inhibitor is a first-line treatment for unresectable or metastatic NSCLC and has been reported to cause fatal non-gastrointestinal fistulas. Respiratory tract fistulas are a known rare complication after bevacizumab therapy characterized by a high mortality rate.

## Introduction

Respiratory tract fistulas (RTFs) are a known rare complication of bevacizumab therapy that are characterized by a high mortality rate [1]. Risk factors associated with this entity include invasive procedures, radiation therapy, and cavitated tumors [1]. RTFs have been reported in patients with primary or metastatic lung cancer [2,3]. Here, we present the formation of a tracheomediastinal fistula in a non-small-cell lung cancer (NSCLC) patient after an endobronchial ultrasound transbronchial needle aspiration (EBUS-TBNA) procedure while on treatment with bevacizumab.

## Case presentation

**Figure 1:**
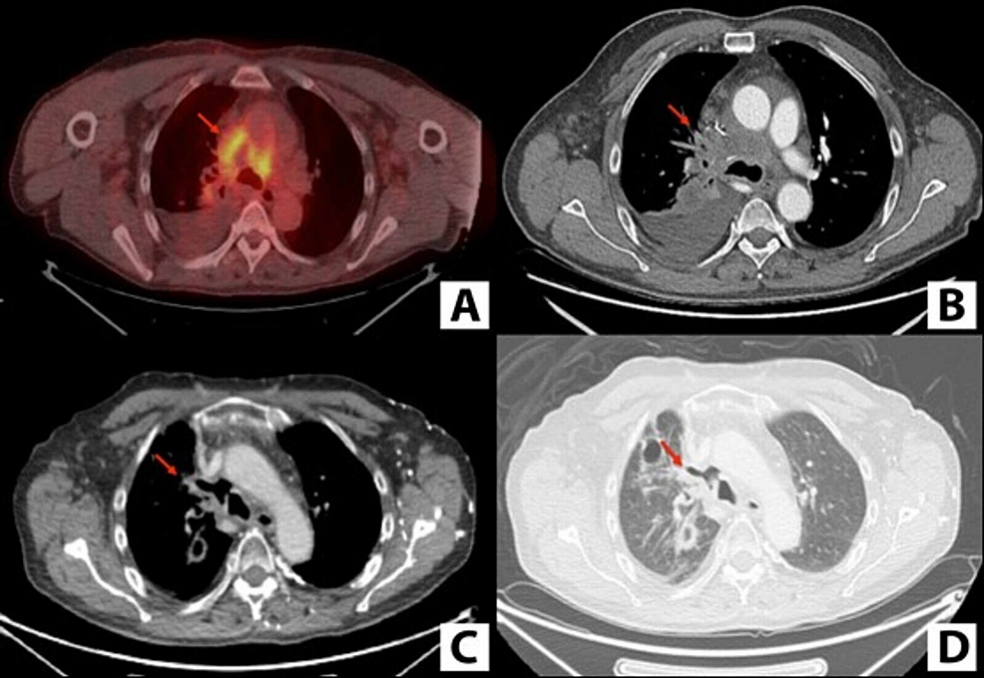
Positron emission tomography showing fluorodeoxyglucose-avid right paratracheal mass (A) and computed tomography showing right paratracheal mass (B). Computed tomography mediastinal window (C) and lung window (D) showing air in the mediastinum.

A 63-year-old man with a 40-pack-year smoking history presented with superior vena cava syndrome secondary to a large right paratracheal mass (Figure 1A, 1B). He was initially seen by a pulmonologist for evaluation of the lung mass and underwent EBUS-TBNA. He was diagnosed with KRAS G12A-mutant metastatic, poorly differentiated, right upper lung NSCLC with mediastinal involvement.

Treatment with definitive chemoradiation with carboplatin, pemetrexed, and pembrolizumab was initiated. After two cycles of chemotherapy, with a mixed response, new metastatic foci were found on imaging scans. He was referred to pulmonary medicine for repeat EBUS-TBNA of the right paratracheal mass to obtain tumor tissue for analysis of molecular markers.

Following the repeat EBUS-TBNA, the patient was started on chemotherapy with carboplatin, paclitaxel, bevacizumab, and atezolizumab every three weeks. Interval imaging demonstrated an overall improvement in metastatic disease, along with a mediastinal air-filled cavity/fistula within the location of the 4R node (Figure 1C, 1D). Because the fistula was a possible result of EBUS-TBNA in the setting of bevacizumab use, bevacizumab was discontinued. Antibiotics were started, and the plan was to monitor the patient in an outpatient setting and repeat imaging in six weeks. Unfortunately, the patient died six days later of massive hemoptysis.

## Discussion

Bevacizumab is a humanized monoclonal antibody that targets vascular endothelial growth factor and thereby inhibits angiogenesis [1-3]. It is a first-line treatment for unresectable or metastatic NSCLC and has also shown promising results for various other cancers, including metastatic colorectal cancer and head and neck cancer [1]. However, several case reports have described an association between the use of bevacizumab and the rare formation of RTFs. RTFs have high morbidity and mortality rates, especially when they involve blood vessels, which can cause hemorrhaging [1-5]. Other adverse effects of bevacizumab include deficient wound healing, proteinuria, hypertension, pneumothorax, diarrhea, both arterial and venous thromboembolic complications, excessive bleeding, gastrointestinal perforations, and reversible posterior leukoencephalopathy syndrome [1-3].

The incidence of RTFs is not well described, but it is believed to be low. However, Kanzaki et al. described that of seven patients who underwent treatment with lung resection and perioperative bevacizumab for metastatic colorectal cancer, two (29%) developed late-onset pulmonary fistulas [3], which suggests that the incidence of RTF may be considerably higher in patients treated with bevacizumab.

Thus far, the reported bevacizumab-associated fistulas have included the following types: malignant tracheal-mediastinal-parenchymal-pleural fistula [1], tracheomediastinal fistula [2], late-onset pulmonary fistula [3], tracheoesophageal fistula [4], and tracheoparenchymal fistula [5]. The associated cancer types include lung, head and neck, and colorectal [1]. The time between exposure to bevacizumab and formation of a fistula varies from several weeks to years after the first dose [2]. The presentation described includes an array of non-specific symptoms such as fever, chills, fatigue, productive cough, and hemoptysis [5].

Risk factors that have been associated with RTF formation after bevacizumab treatment include recent invasive procedures such as EBUS and surgery, damage to esophageal and respiratory tract structures after chemotherapy and/or radiation therapy, squamous cell carcinoma, central location and cavitation of the tumor [1-5], superimposed infection [5], and large subcarinal lymph nodes [4]. However, Thawani et al. stated that these factors are not imperative as fistula formation has occurred in patients with no risk factors beyond the use of bevacizumab [2]. On the other hand, Alzghoul and Meena described that fistulas can form even without bevacizumab when concurrent chemoradiation therapy is used [5].

Although the definite pathophysiology of RTF is unknown, it has been hypothesized that impaired wound healing and vessel formation secondary to bevacizumab can lead to the formation of fistulas after damage to the mucosa [1]. Some have suggested waiting six to eight weeks after the last dose of bevacizumab before performing surgery and delaying initiation of bevacizumab postoperatively for at least 28 days [3,6].

Fistulas affecting the airway have been managed in various ways based on the patient’s individual situation [2]. Case reports describe the use of surgery, endoscopy, and flaps to close and repair the fistula. Additionally, autologous stem cells derived from adipose tissue, plasma coagulation, and fibrin glue have also been used to repair the damage caused by fistulas [1,2].

## Conclusions

In our patient’s case, we believe that both the two cycles of bevacizumab and the chemoradiation therapy he received contributed to the formation of the fistula. The reported literature about bevacizumab-associated RTFs continues to lack detail. Therefore, it is important to analyze all patients with RTFs as a group to better understand the pathophysiology of this complication, its true incidence and prevalence, and what preventive measures should be taken. Follow-up of the resolved fistulas is needed to determine the long-term outcomes and whether any specific treatment has advantages over the others.
